# Mechanisms and Effectiveness of Online CB-ART Interventions in Reducing Covid-19-related Distress

**DOI:** 10.1192/j.eurpsy.2022.509

**Published:** 2022-09-01

**Authors:** D. Segal-Engelchin, V. Daitchman, E. Huss, O. Sarid

**Affiliations:** Ben-Gurion University of the Negev, Social Work, Beer-Sheva, Israel

**Keywords:** Image transformations, Covid-19, Distress, CB-ART interventions

## Abstract

**Introduction:**

The combination of cognitive behavioral interventions and art therapy provides a unique tool for image transformation as a strategy for managing distress in extremely stressful situations. Previous studies offer evidence of the effectiveness of cognitive behavioral- and art-based (CB-ART) interventions in reducing stress related to community crises such as wars and earthquakes.

**Objectives:**

This study aimed to extend current knowledge by investigating the effectiveness of CB-ART interventions in the Covid-19 context, and the mechanisms underlying them.

**Methods:**

Online CB-ART interventions were implemented during the first national lockdown in Israel with 15 women. The intervention included drawing three pictures related to: (1) Covid-19-related emotions and thoughts; (2) resources that may help them cope with the pandemic outcomes; and (3) integration of the stressful image and the resource picture. To examine the intervention effect, participants’ Subjective Units of Distress (SUDs) values were measured using a pre-post design.

**Results:**

Participants’ initial distress levels decreased on completion of the intervention. Another key finding is the reduction of the initial size of the stressful image and enlargement of the resource images within the integrated drawing. This may be the proposed mechanisms underlying the reduction of the SUDs values.

**Conclusions:**

The new perspective derived from the compositional transformations performed by the participants may have increased their sense of control and competence, enabling them to perceive the Covid-19-related stressors as less threatening. The described art-based tool can be easily implemented online by mental health professionals with diverse populations in times of community crises.

**Disclosure:**

No significant relationships.

